# Regional differences in the reduction in cerebral FDG uptake induced by the ketogenic diet

**DOI:** 10.1186/s41824-022-00150-5

**Published:** 2022-12-15

**Authors:** O. A. Bennett, S. C. Ramsay, E. Malacova, P. Bourgeat, S. J. Goodman, C. J. Dunn, B. M. Robinson, K. Lee, D. A. Pattison

**Affiliations:** 1 Department of Nuclear Medicine & Specialised PET Services, Royal Brisbane & Women’s Hospital, Brisbane, Australia; 2 Nuclear Medicine and PET/CT Department, Prince of Wales Hospital, Sydney, Australia; 3 QIMR Berghofer Medical Research Institute, Brisbane, Australia; 4 Australian E-Health Research Centre, CSIRO Health and Biosecurity, Brisbane, Australia; 5 School of Medicine, University of Queensland, Brisbane, Australia

**Keywords:** FDG PET, Ketosis, Cerebral glucose metabolism, Alzheimer’s disease, Dementia

## Abstract

**Background:**

The postulated benefits of the ketogenic diet in the management of multiple medical conditions have seen more patients who are in therapeutic ketosis attending ^18^F-FDG PET scans. This study aimed to investigate the effect of ketosis on cerebral glucose metabolism in a clinical PET scanning environment using ^18^F-FDG uptake as a surrogate marker.

**Methods:**

A retrospective audit was conducted of the brain ^18^F-FDG uptake in 52 patients who underwent PET scans for possible cardiac sarcoidosis or suspected intracardiac infection, following a ketogenic diet and prolonged fasting. SUVbw for whole brain and separate brain regions was compared with serum glucose and serum ketone body (beta-hydroxybutyrate) levels.

**Results:**

The expected negative association between serum glucose levels and whole brain ^18^F-FDG uptake was confirmed. A reduction in SUVbw due to increasing serum ketones levels was also observed that was independent of and in addition to the effects of glucose. The magnitude of the reduction in SUVbw related to serum glucose level and serum ketone level was found to be greater in the precuneus than in the cerebellum or whole brain.

**Conclusion:**

In a real-world clinical PET setting, cerebral ^18^F-FDG uptake appears to be affected by glycaemia and ketonaemia. This means when assessing the brain, both serum glucose and ketone levels need to be considered when SUVs are used to distinguish between pathologic and physiologic states. The magnitude of this effect appears to vary between different brain regions. This regional difference should be taken into consideration when selecting the appropriate brain region for SUV normalisation, particularly when undertaking database comparison in the assessment of dementia.

## Background

The ketogenic diet has experienced a renaissance in recent years due to its role in the management of paediatric refractory epilepsy. Research is also amassing on its use in the management of refractory epilepsy in adults, as well as in neurodegenerative disorders including Alzheimer’s disease (AD), hypoxic and hypoglycaemic brain injury, and oxidative damage by reactive oxygen species and free radicals (Bough et al. 2006; Gasior et al. 2006; Kashiwaya et al. 2000; Kim et al. 2007; Maalouf et al. 2007; Samoilova et al. 2010). Numerous studies have indicated cognitive improvements in patients with AD given a ketogenic diet (Brandt et al. 2019; Rubia Ortí et al. 2018; Taylor et al. 2018). Ketogenic diets can assist in the management of type 2 diabetes (Dashti et al. 2007; Yuan et al. 2020), and there is extensive literature examining the postulated benefits of ketogenic diets in malignancy (Sremanakova et al. 2018).

Although these published studies support the potential benefits of a ketogenic diet in many disorders, its use remains contentious in a number of these conditions and more research is required. For example, the mechanism by which the ketogenic diet imparts its benefits in neurological conditions is not completely understood. Proposed mechanisms include simple models in which ketone bodies act as alternate fuel substrates to glucose and more complex models involving the alteration of intracellular metabolic pathways (Bough et al. 2006; Gasior et al. 2006; Kashiwaya et al. 2000; Maalouf et al. 2009).

Because of this range of proven and postulated benefits of ketosis, more patients are attending 2-[^18^F]fluoro-2-deoxy-D-glucose (^18^F-FDG) positron emission tomography (PET) of the brain while in therapeutic ketosis. Given that ketones can act as a metabolic substrate for the brain, it is relevant to understand the impact of ketosis on these images. Patients referred to our department for ^18^F-FDG PET evaluation of cardiac inflammation undertake a strict ketogenic diet for 2 days prior to the study, followed by an 18-h fast. Blood ketone levels are measured as part of our documentation of adherence to this dietary preparation which renders many of our patients ketotic (Robinson et al. 2018). Consequently, the relationship between ^18^F-FDG uptake of the brain in these patients and their blood glucose and ketone levels was performed to explore the effects of ketosis on ^18^F-FDG uptake in the brain in a real-world clinical PET scanning environment.

## Methods

### Patients

A retrospective audit of ^18^F-FDG PET scans performed over an 8-month period from August 2019 to April 2020 on 72 consecutive patients for the indication of possible cardiac sarcoidosis or suspected intracardiac infection was undertaken. Fifteen patients were excluded as their brains were not imaged or were incompletely imaged. Four were excluded as they had prior cortical infarcts with regions of hypometabolism on ^18^F-FDG PET. An additional patient was excluded as their images were corrupted during spatial transformation. None of the included patients had a clinical history of dementia, or clinical or scan evidence of active cerebral sarcoidosis.

Fifty-two patients were included in the final analysis: 37 male (71%) and 15 female (29%). The males ranged in age from 32 to 86 years (mean age = 56.7 years) and the females from 36 to 77 years (mean age = 59.2 years). Thirteen patients (25%) were diabetic (eight male, five female), and of these, twelve had type 2 diabetes mellitus (T2DM) and one had type 1 diabetes mellitus (T1DM). Five (10%) patients were on regular insulin, including the single patient with T1DM. The male and female patients had comparable demographics although the male patients were significantly heavier and taller than the female patients. (More detailed demographic data are included in Table 1).

**Table 1:** Patients’ demographics

Pt No.	Age	Gender	Weight	Height	T1DM/T2DM	Oral hypoglycaemic	Insulin	Dietary adherence	Fasting period	Serum ketones	In ketosis?	Urinary ketones	Blood glucose	Heparin (50U/kg, max 5000U)	Injected activity (MBq)	Uptake period (min)
2	66	M	115.8	186	–	–	–	Yes	20.1	0.6	Yes	Not done	5.3	No	314	68
3	62	M	115.1	188	–	–	–	Yes	23.5	0.6	Yes	Negative	5.2	No	331	95
4	61	F	102	157	T2DM	Metformin	No	Yes	19.9	0.6	Yes	Negative	5.9	No	310	76
6	78	M	98.3	172	–	–	–	Yes	18.2	0.9	Yes	3.9	5.1	No	299	60
7	86	M	70.4	171	–	–	–	Yes	18.9	1.2	Yes	1.5	4.4	No	289	78
9	53	F	64.4	164	–	–	–	No	19.9	0.7	Yes	Negative	4.6	No	266	58
10	66	F	76	162	T2DM	Metformin, Gliclazide	No	Yes	20.7	0.4	No	Negative	9.3	3800	282	64
12	51	F	80.6	162	–	–	–	Yes	19.3	1.1	Yes	3.9	4.3	4000	313	66
13	48	M	71.6	167	–	–	–	Yes	18.8	0.4	No	Negative	5.5	3600	307	70
14	76	M	112.7	176	–	–	–	Yes	19.5	1.3	Yes	3.9	4.4	5000	323	60
15	36	F	79.8	168	–	–	–	Yes	20.9	0.8	Yes	1.5	5.4	4000	300	60
16	74	M	82.9	176	–	–	–	Yes	19.6	0.7	Yes	1.5	5.6	No	317	71
17	60	M	84	179	–	–	–	Yes	21.3	1.1	Yes	3.9	4.3	No	319	72
18	60	F	78.1	157	T2DM	Metformin, Dapagliflozin	25 U mane	Yes	19.0	1.7	Yes	3.9	5.3	3900	307	71
19	38	M	115.2	176	–	–	–	Yes	20.8	0.6	Yes	3.9	4.4	5000	301	70
22	49	M	107.7	184	–	–	–	Yes	25.5	0.8	Yes	1.5	5.7	5000	338	63
23	59	M	117	176	–	–	–	Yes	19.4	0.3	No	Negative	5.6	5000	265	57
24	47	M	73.5	174	–	–	–	Yes	18.2	0.9	Yes	1.5	3.6	No	306	78
25	32	M	129.3	167	–	–	–	Yes	19.8	0.3	No	Negative	5.3	No	313	73
26	55	M	125.4	171	T2DM	–	Lantus, Novorapid	Yes	19.0	0.2	No	Negative	7.7	5000	317	68
27	59	M	73	173	–	–	–	Yes	19.9	0.8	Yes	1.5	4.4	3650	301	61
28	46	M	114	176	T2DM	Metformin, Gliclazide, Sitagliptin	No	No	19.1	0.7	Yes	Not done	7.5	5000	282	66
29	59	M	85.5	175	–	–	–	Yes	18.8	0.7	Yes	Not done	4.1	No	322	66
30	62	F	69	157	–	–	–	Yes	19.3	2.5	Yes	> 15.6	3.9	No	307	60
31	40	M	88	175	–	–	–	Yes	20.3	0.8	Yes	3.9	4.6	4400	309	64
32	40	M	71	172	–	–	–	No	19.3	2.1	Yes	3.9	4.4	3500	304	58
33	48	F	75.5	170	–	–	–	Yes	19.4	0.7	Yes	Negative	4.2	No	322	68
34	50	M	116	178	–	–	–	Yes	17.0	0.8	Yes	Not done	4.9	5000	314	60
36	76	M	79.8	168	–	–	–	Yes	22.5	1.3	Yes	3.9	4.9	4000	302	57
37	60	M	105	182	–	–	–	Yes	21.2	0.5	Yes	Trace	4.7	5000	287	60
38	75	F	105.9	163	–	–	–	Yes	16.7	0.3	No	Negative	4.8	No	225	86
39	67	M	99.5	175	T2DM	Metformin, Vildagliptin, Empagliflozin	No	No	7.0	3.8	Yes	3.9	9.7	No	290	74
40	55	M	91.3	178	–	–	–	Unknown	22.0	0.8	Yes	3.9	4.4	4500	330	76
41	77	F	80.3	154	T2DM	Metformin	No	Yes	20.3	0.5	Yes	Negative	7.1	No	330	
42	63	M	94.5	189	–	–	–	Yes	24.1	0.5	Yes	Negative	5.8	No	309	70
43	55	M	83.2	162	T2DM	Metformin, Empagliflozin	No	Yes	19.3	1.9	Yes	3.9	8.4	No	309	62
44	58	F	89.7	164	–	–	–	Yes	22.1	1.8	Yes	7.8	4.3	4650	290	50
45	57	M	163	182	T2DM	Metformin, Sitagliptin	No	Yes	20.2	0.3	No	Negative	11.7	5000	302	108
46	35	M	79.1	185	T1DM	No	Lantus, Novorapid	Yes	21.6	0.3	No	Negative	4.1	4000	307	67
48	45	M	107.8	180	–	–	–	Yes	19.2	0.6	Yes	Trace	6.1	5000	310	60
49	33	M	95.2	172	–	–	–	Yes	19.6	0.3	No	Negative	5.4	No	309	78
50	76	F	75.1	168	T2DM	No	Novomix	Yes	18.7	1.6	Yes	Negative	8.9	No	310	87
51	66	M	90.4	162	–	–	–	Yes	19.1	0.8	Yes	Trace	5.8	No	311	64
53	56	F	63.6	156	–	–	–	Yes	19.3	1.7	Yes	Negative	4.8	3250	273	77
58	62	M	130	161	T2DM	Metformin	Novorapid, Lantus	No	19.0	1.1	Yes	Trace	9	No	331	59
60	66	F	72	163	–	–	–	Yes	18.8	0.3	No	Negative	5.9	No	276	57
61	47	M	75.4	174	–	–	–	Yes	19.5	0.6	Yes	Negative	4.9	3750	309	62
63	82	M	75.1	161	–	–	–	Yes	20.8	1.6	Yes	Not done	4.3	No	300	64
66	59	M	98	179	–	–	–	Yes	17.0	0.4	No	Negative	5.5	4900	313	86
67	44	F	74.8	160	–	–	–	Yes	N/A	0.8	Yes	Trace	6.2	3700	309	81
70	64	M	94.5	170	T2DM	Metformin	No	Yes	15.0	0.4	No	Negative	5.3	No	309	61
72	73	M	44	162	–	–	–	Yes	17.7	5.4	Yes	1.5	2.4	No	184	74

### Diet

All patients received a letter outlining the required dietary preparation. Subsequently, the dietary preparation was explained to patients via a combination of in-person consultation with one of our nuclear medicine physicians, via email and/or via telephone call. Permissible and impermissible food items were outlined (Table 2) with emphasis made on the importance of strict dietary adherence. The patients were asked to commence the prescribed ketogenic diet for 48 h and commence fasting for 18 h prior to their scheduled ^18^F-FDG PET. Diabetic patients were asked to withhold oral hypoglycaemic medications and subcutaneous insulin during the 18-h fast period. The T1DM patient was asked to consult their endocrinologist prior to commencing the ketogenic diet and asked to withhold insulin for at least 4 h prior to their ^18^F-FDG injection.

**Table 2:** Preparatory diet

*Permitted foods (high fat/protein permitted, low carbohydrate)*
Fried fatty, unsweetened meats (i.e. chicken, turkey, fish, steak, ham, bacon)
Fried eggs
Tea and coffee without milk or sugar
Water
Sugar substitutes (e.g. Equal, Nutrasweet, Sweet’N Low)
*Foods not permitted*
Food containing carbohydrates and sugars including artificial sweeteners containing sucralose (e.g. Splenda) and lactose (e.g. sugarless)
Processed meats (including sausages), milk, cheese, bread, bagels, cereal, cookies, toast, pasta, crackers, muffins, peanut butter, nuts, fruit juices, potatoes, lollies, fruit, rice, chewing gum, mints, cough drops, vegetables, beans and alcohol

On the day of the study, the patients self-reported adherence to the preparatory diet including the time and constitution of their last meal. The duration of the fasting period was calculated from the time of the last meal to the time of the ^18^F-FDG injection.

### Blood glucose and ketone measurement

Patients’ blood ketone levels and blood glucose levels (BGL) were measured prior to ^18^F-FDG injection. Ketone levels were measured using the FreeStyle Optium Neo Ketone monitoring system (Abbott Diabetes Care, UK) which measures beta-hydroxybutyrate (BHB). Typically, BHB accounts for 78% of circulating ketone bodies, while acetoacetate and acetone account for 20% and 2% of circulating ketone bodies, respectively (Laffel 1999); hence, BHB is a suitable marker of an individual’s ketotic state. Patients with serum BHB levels ≥ 0.5 mmol/L were deemed to be in ketosis, and those with levels < 0.5 mmol/L were deemed not ketotic (Laffel 1999). BGL was measured using the FreeStyle Optium Neo H monitoring system (Abbott Diabetes Care, UK).

### ^18^F-FDG PET/CT

Following ^18^F-FDG injection, the patients were reclined in a quiet, dimly lit room for 50–108 min (mean = 69 min). Injected activity of ^18^F-FDG ranged from 184 MBq (0.005 Ci) to 338 MBq (0.009 Ci), mean = 308 MBq (0.008 Ci). As part of our protocol to suppress myocardial uptake, 27 patients (52%) received unfractionated heparin intravenously (50 IU/kg to a maximum of 5000 IU) 15 min prior to the injection of ^18^F-FDG.

PET/CT images were acquired from vertex to thighs using the Siemens Biograph mCT or Siemens MCT Flow for 2 min/bed position with patients supine. Most of our patients were scanned with their arms elevated above their head to avoid beam-hardening artefacts through the torso, although a few patients were scanned with their arms down by their sides due to physical limitations. Patients were instructed to breathe normally throughout their scans. The CT acquisition consisted of a CT topogram and low-dose non-contrast CT acquisition covering the same region.

The PET/CT images were reconstructed using standard algorithms and displayed in sagittal, coronal, and transverse planes using Siemens Syngo.via.

The whole body effective dose ranged from 3.5 mSv to 6.4 mSv (mean 5.9 mSv) for the ^18^F-FDG PET and from 3 to 5 mSv for the CT. Total whole body effective doses ranged from 6.5 mSv to 11.4 mSv (i.e. ^18^F-FDG PET plus CT). Patients were asked to void immediately prior to their scans to reduce radiation doses.

### Image analysis

PET scans were analysed in Siemens Syngo.via MI Neurology and MM Oncology. Using MI Neurology, the brains were spatially transformed into the Montreal Neurosciences Institute brain space. SUVmean values normalised for bodyweight (SUVbw) were then calculated for volumes of interest using the tabulated values in the MI Neurology protocol “Analysis” tool for whole brain, precuneus, basal ganglia and cerebellum.

PET images were further analysed using CapAIBL, a PET quantification software which allows the mapping of SUV values onto a template surface mesh for voxel-based analysis (Zhou et al. 2014).

Liver and blood pool values were calculated using the MM Oncology automated “Liver Reference Region” and “Aorta Reference Region” tools, respectively. For 2 studies (4%), the volume-of-interest (VOI) for blood pool (i.e. “Aorta Reference Region”) was manually repositioned due to unsatisfactory alignment with the descending aorta due to anatomic variability.

### Statistical analysis

Using unadjusted and adjusted linear regression models, we explored the relationship between SUVbw measurement within the whole brain, and BGL and serum ketones.

We then applied the same models on a voxel-by-voxel basis using CapAIBL displaying the resultant t-scores and the magnitude of the coefficients in colour-coded parametric brain surface maps.

To further evaluate the relationship between SUVbw of the precuneus and the whole brain with BGL and serum ketones, we fitted linear mixed-effects models for repeated measures. We also fitted linear mixed-effects models for repeated measures to evaluate the relationship between SUVbw of the precuneus versus cerebellum compared to BGL and serum ketones.

For all models, we explored two-way interaction terms for all significant variables, and statistical significance was set at the 5% level (two-sided), which has been corrected for multiple comparisons using the false discovery rate for the voxel-by-voxel basis analysis.

All analyses were performed using MedCalc and STATA version 15.1 (Stata Corporation, College Station, Texas, USA).

## Results

### Diet

The fasting period ranged from 7 to 25.5 h (mean = 19.5 h). Seventy patients reported fasting times of 15 h or longer, while one patient did not have fasting time documented.

### Blood glucose and ketone measurement

BGL ranged from 2.4 to 11.7 mmol/L (mean 5.7 mmol/L), and serum ketone levels ranged from 0.2 to 5.4 mmol/L (mean 1 mmol/L). In our department, BGL is usually required to be < 10.0 mmol/L for an ^18^F-FDG PET to proceed. One patient exceeded this with a BGL of 11.7 mmol/L. Their ketone level was 0.3 mmol/L. This patient confirmed adherence to the prescribed diet, and the hyperglycaemia was attributed to their T2DM. A clinical decision was made to proceed with the scan. Five patients (10%) stated that they did not strictly adhere to the prescribed diet, and dietary adherence was unknown for one patient, but all six of these patients were found to be in ketosis. Twelve patients (23%) were not in ketosis (i.e. they had serum ketones < 0.5 mmol/L), but all these patients reported strict compliance with the dietary preparation and were included in our analysis.

### ^18^F-FDG PET/CT

**Fig. 1:**
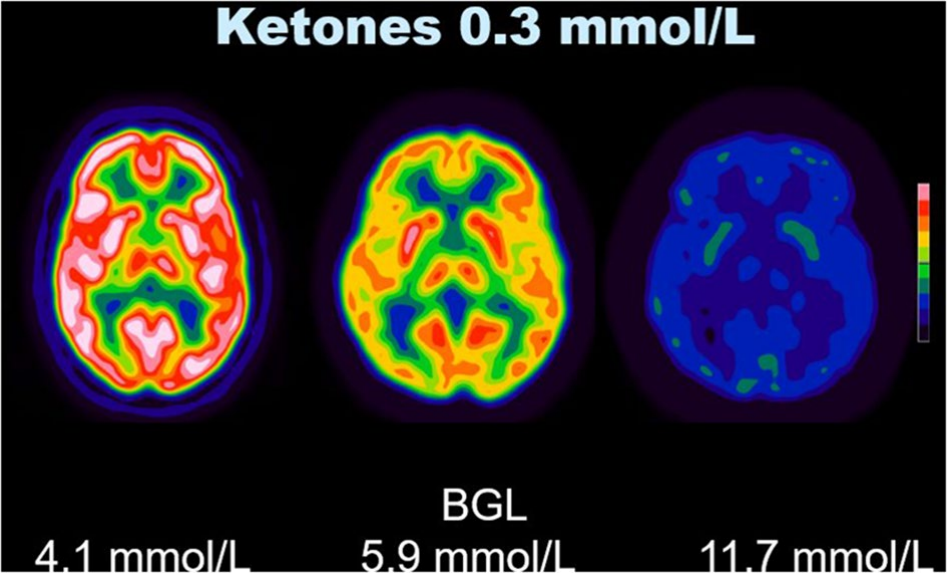
Effect of BGL on cerebral SUVbw. Sample images of three patients thresholded identically with the same serum ketone level, but different BGLs illustrate the sometimes dramatic reduction in FDG uptake in the brain that can accompany a high BGL

**Fig. 2:**
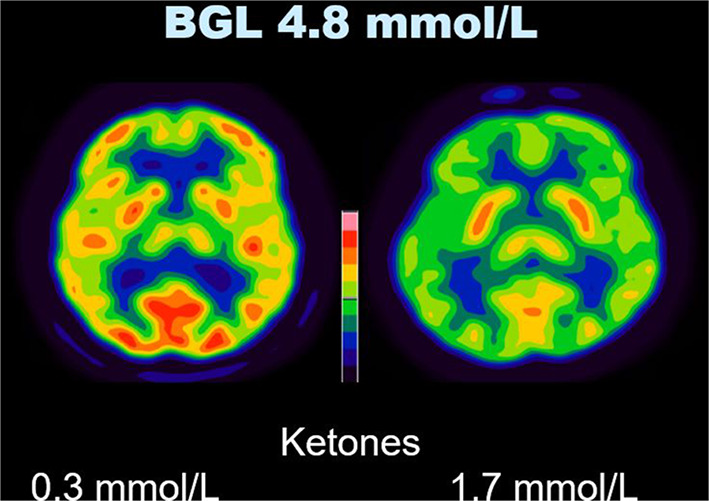
Effect of serum ketone level on cerebral SUVbw. Sample images of two patients thresholded identically with the same BGL, but different serum ketone levels illustrate the reduction in FDG uptake in the brain seen with a higher serum ketone level

In unadjusted analysis of the whole brain, BGL was strongly negatively associated with SUVbw (*t* = − 4.41, *p* < 0.001), while serum ketones had a weaker association with SUVbw (*t* = − 2.38, *p* = 0.02) (Figs. 1 and 2). After mutual adjustment, both BGL (*t* = − 5.28, *p* < 0.001) and serum ketones (*t* = − 3.56, *p* < 0.001) remained negatively associated with SUVbw (Table 3). The inclusion of an interaction term of BGL with serum ketones did not significantly improve the model, indicating that BGL and serum ketones do not interact in their association with SUVbw. Adding age to the adjusted model did not further improve its fit. Brain SUVbw was not found to be affected by the administration of intravenous heparin or the residual amount of ^18^F-FDG present in the blood (as determined by mediastinal blood pool SUVbw) at the time of scanning. When the adjusted linear regression model was applied to SUVbw for the cerebellum and basal ganglia, the results confirmed that the uptake of FDG within these regions was also suppressed by increases in both BGL and serum ketone levels (Tables 4 and 5).

**Table 3:** Adjusted linear regression analysis of whole brain SUVbw against BGL and serum ketones

Parameter	Coef	Standard error	*T*	P*
BGL	− 0.71	0.13	− 5.28	< 0.0001
Serum ketones	− 0.92	0.26	− 3.56	0.0008
Constant	12.04			

*Statistical significance (*P* < 0.05)

**Table 4:** Adjusted linear regression analysis of cerebellum SUVbw against BGL and serum ketones

Parameter	Coef	Standard error	*t*	P*
BGL	− 0.60	0.12	− 5.12	< 0.0001
Serum ketones	− 0.66	0.22	− 2.94	0.005
Constant	10.55			

*Statistical significance (*P* < 0.05)

**Table 5:** Adjusted linear regression analysis of basal ganglia SUVbw against BGL and serum ketones

Parameter	Coef	Standard error	*t*	P*
BGL	− 0.84	0.16	− 5.19	< 0.0001
Serum ketones	− 1.10	0.32	− 3.52	0.0009
Constant	13.74			

*Statistical significance (*P* < 0.05)

**Fig. 3:**
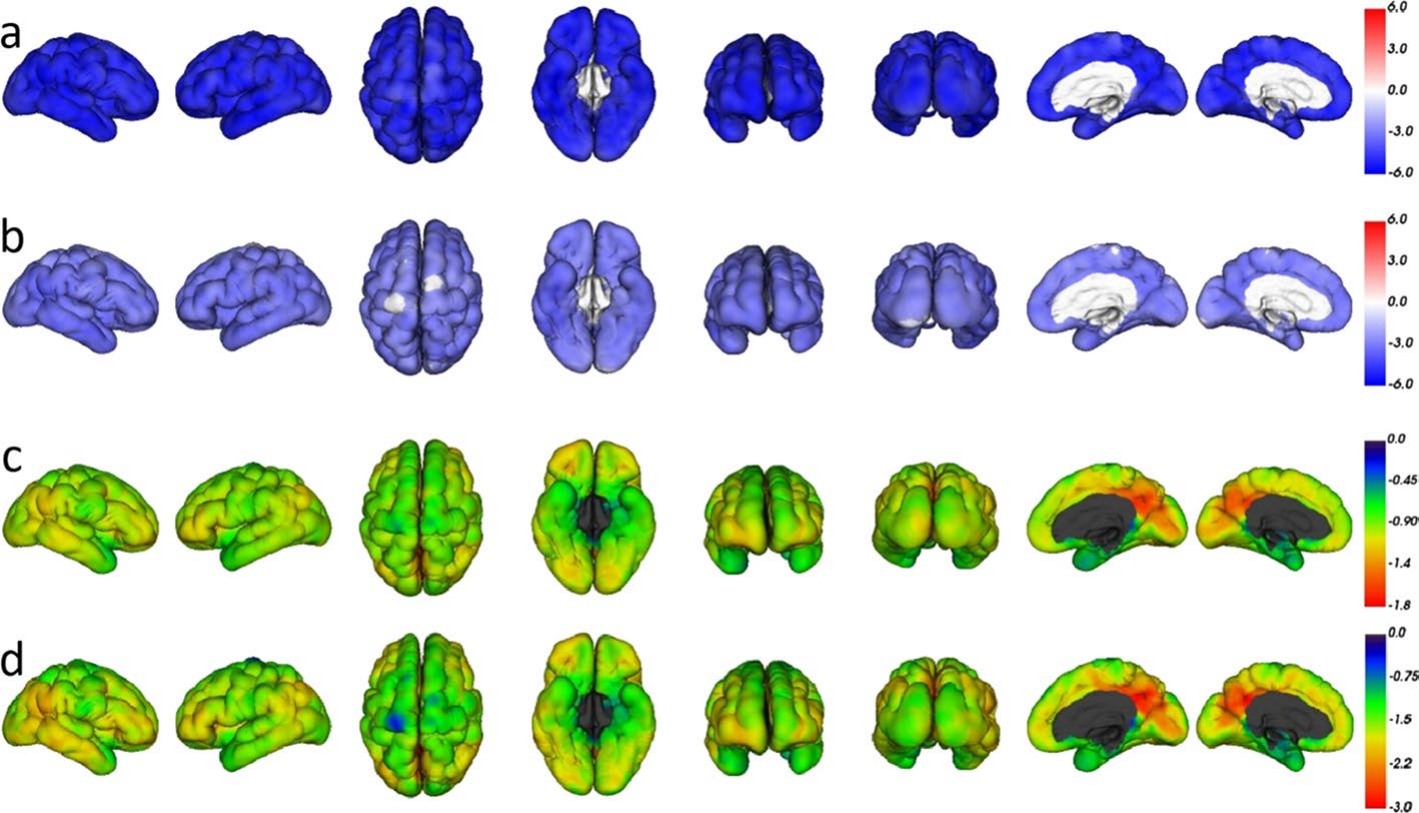
Adjusted linear regression analysis of SUVbw against BGL and serum ketones performed on a voxel-by-voxel basis and projected onto a cortical surface map to aid localisation using CapAIBL. **a** t-score map for significance of change in SUVbw related to BGL. The strongly negative t-scores (deep blue) across the entire cortex indicate increasing BGL suppresses FDG uptake across the entire cortex; **b** t-scores for serum ketones are also significant across the entire cortex, confirming that higher serum ketones suppress SUVbw in addition to the suppressive effect of increasing BGL; **c** plot of the value of the coefficient for BGL in the multiple linear regression equation for each voxel, showing that this coefficient has greater magnitude (red) in the region of the precuneus; **d** plot of the value of the coefficient for serum ketones, showing that this also has greatest magnitude in the precuneus

When this adjusted linear regression model was applied on a voxel-by-voxel basis using CapAIBL, BGL and serum ketones were found to suppress SUVbw across the entire cerebral cortex (Fig. 2). The coefficients for BGL and serum ketones were of greater magnitude in the precuneus than they were in other regions of the cerebral cortex (Fig. 3).

#### Precuneus versus whole brain

We then compared SUVbw in the precuneus with that in the whole brain (Table 6). We found that precuneus SUVbw was positively associated with whole brain SUVbw (*p* < 0.001). When adjusting for brain region, BGL (*p* < 0.001), as well as serum ketones (*p* = 0.016), was negatively associated with SUVbw. Their mutual adjustment showed little change in their association between BGL (*p* < 0.001) and serum ketones (*p* < 0.001) and SUVbw.

**Table 6:** Mixed-effects model of precuneus against whole brain, including two-way interactions

Parameter	Coef	*z*	P*	Lower 95% CI	Upper 95% CI
Region	3.66	10.23	0.000	2.96	4.37
BGL	− 0.71	− 4.51	0.000	− 1.02	− 0.40
Region*BGL	− 0.28	− 4.96	0.000	− 0.39	− 0.17
Serum ketones	− 0.92	− 3.04	0.002	− 1.51	− 0.33
Region*serum ketones	− 0.34	− 3.16	0.002	− 0.55	− 0.13
Constant	12.04	12.09	0.000	10.09	13.99

*CI* confidence interval

*Statistical significance (*P* < 0.05)

There were two significant interaction terms with brain region. The first interaction term between BGL and brain region suggests that for each 1 mmol/L increase in BGL there was a corresponding decrease in SUVbw of 0.71 (*p* < 0.001) in the whole brain. There was a more pronounced effect in the precuneus brain region, where each 1 mmol/L increase in BGL was associated with an additional decrease in SUVbw of 0.28 (0.99 total decrease per 1 mmol/L increase in BGL). At lower BGL, the SUVbw in the precuneus was significantly higher than the whole brain SUVbw. This difference in SUVbw decreased as BGL increased until BGL reached a maximum value of 12 mmol/L at which point there was no difference in SUVbw in the precuneus compared to the whole brain.

A similar picture was observed for the second interaction term between serum ketones and brain region, which suggests that for each 1 mmol/L increase in serum ketones there was a corresponding decrease in SUVbw of 0.91 (*p* = 0.002) in the whole brain with an additional decrease in SUVbw in the precuneus of 0.34 (1.25 total decrease per 1 mmol/L increase in serum ketones). When the serum ketone level was extrapolated to 6 mmol/L (5.4 mmol/L was the maximum value in our cohort), there was no difference between SUVbw in the precuneus and the whole brain.

#### Precuneus versus cerebellum

When comparing the precuneus to the cerebellum in unadjusted analysis, the precuneus brain region was positively associated with SUVbw (*p* < 0.001). In our more complex mixed-effects model (Table 7), after adjustment for brain region, BGL (*p* < 0.001), as well as serum ketones (*p* = 0.025), was negatively associated with SUVbw. After mutual adjustment, BGL (*p* < 0.001) and serum ketones (*p* = 0.023) remained negatively, and brain region (*p* < 0.001) was positively associated with SUVbw.

**Table 7:** Mixed-effects model of precuneus against cerebellum, including two-way interactions

Parameter	Coef	*z*	P*	Lower 95% CI	Upper 95% CI
Region	5.15	9.56	0.000	4.10	6.21
BGL	− 0.60	− 3.98	0.000	− 0.89	− 0.30
Region*BGL	− 0.39	− 4.61	0.000	− 0.56	− 0.23
Serum ketones	− 0.657	− 2.28	0.023	− 1.22	− 0.09
Region*serum ketones	− 0.60	− 3.68	0.000	− 0.62	− 0.28
Constant	10.55	11.08	0.000	8.69	12.42

*CI* confidence interval

*Statistical significance (*P* < 0.05)

For this comparison, there were two significant interaction terms with brain region (both *p* < 0.001). The interaction term between BGL and brain region suggests that for each 1 mmol/L increase in BGL there was a corresponding decrease in SUVbw of 0.51 in the cerebellum with an additional decrease in SUVbw in the precuneus of 0.39 (0.90 total decrease per 1 mmol/L increase in BGL). There were significantly higher SUVbw values in the precuneus compared to the cerebellum at lower BGL with this difference decreasing as BGL increased. Once BGL reached 12 mmol/L, there was no difference in SUVbw between the precuneus and cerebellum.

The interaction term between serum ketones and brain region suggests that for each 1 mmol/L increase in serum ketones in the cerebellum there was a corresponding decrease in SUVbw of 0.66, and there was an additional 0.60 decrease in SUVbw in the precuneus (1.26 total decrease per 1 mmol/L increase in serum ketones). Thus, the originally higher SUVbw in the precuneus decreased more rapidly than the SUVbw in the cerebellum until serum ketones reached 5 mmol/L at which point there was no difference in SUVbw between the precuneus and cerebellum. However, when serum ketones increased further still to 5.4 mmol/L (the maximum level in our cohort), the cerebellum had a slightly higher SUVbw than the precuneus.

No significant association was found between brain ^18^F-FDG uptake and the level of blood pool activity or the administration of intravenous heparin.

## Discussion

This study confirms the expected significant negative association between BGL and brain ^18^F-FDG uptake that has been identified in multiple prior studies (Ishibashi et al. 2017; Sarikaya et al. 2019; Viglianti et al. 2017). This observed effect is thought to be most likely due to competitive inhibition of ^18^F-FDG uptake by glucose at glucose transporters (GLUTs). Viglianti et al. (Viglianti et al. 2017) previously suggested that variation in serum glucose levels affects ^18^F-FDG uptake in a nonlinear/dual linear fashion due initially to saturation of GLUTs followed by saturation of intracellular hexokinase at higher serum glucose levels with a threshold of 125 mg/dl (i.e. 6.9 mmol/L). However, when we compared three possible models with and without transformation ((a) a linear model with log-transformed outcome variable, (b) a generalised linear model with log link function, and (c) a linear model with no transformation), we found that the linear model with no transformation had a comparable overall model fit to the other two more complex models, thus justifying the choice of the simpler model that we have used for our analyses. It is possible that this finding may be due to the majority of our patients’ BGLs falling within the euglycaemic range in which ^18^F-FDG uptake is still determined by the level of GLUT saturation.

We also found that increasing serum ketone levels have a suppressive effect on brain ^18^F-FDG uptake that is additional to, and independent of, the suppressive effect of BGL. Suppression of ^18^F-FDG brain uptake during ketosis has been observed previously in humans using PET scans under experimental conditions (Courchesne-Loyer et al. 2017; Cunnane et al. 2016), but to the best of our knowledge, this is the first time it has been found to have a measurable effect on ^18^F-FDG PET scans performed for clinical purposes. Although brain SUVbw varied with both serum glucose and serum ketone levels, it is interesting to note that in our patient group the effect of ketones was partially masked by BGL, being found to be more obvious once the relationship between BGL and ^18^F-FDG uptake has been accounted for. This complexity may explain why the suppression of brain ^18^F-FDG uptake due to ketosis has not previously been recognised in clinical scans.

The reduction we have found in ^18^F-FDG uptake associated with ketones most likely reflects a true reduction in glucose metabolism by the brain due to the preferential use of ketone bodies as an alternate energy substrate independent of glucose availability. Ketone bodies are a more efficient source of adenosine triphosphate (ATP) production per unit of oxygen than glucose (Gasior et al. 2006; Courchesne-Loyer et al. 2017; Cunnane et al. 2016; Veech et al. 2001; Hasselbalch et al. 1995; LaManna et al. 2009). Our analysis indicates that the reduction in brain ^18^F-FDG uptake associated with increasing ketone levels can be modelled as a straight line so long as BGL is simultaneously considered. It has previously been shown that cerebral ketone uptake increases linearly with increasing serum ketone concentrations (Cunnane et al. 2016) and that an inverse relationship exists between brain glucose and ketone metabolism in normal adults during short-term moderate dietary ketosis (Courchesne-Loyer et al. 2017), leading those authors to propose that overall cerebral metabolic rate (CMR) is a sum of CMRketones + CMRglucose.

These findings have direct clinical implications when SUV thresholds are used to help differentiate between pathologic and physiologic processes (e.g. comparing pathologic uptake in glioma to that of normal brain tissue), suggesting that SUVs may need to be corrected for serum ketone levels as well as BGL in such situations.

Our results indicate that BGL and serum ketones suppress ^18^F-FDG uptake in all areas of the brain. However, we found that for both BGL and ketones, the degree of suppression is not uniform in all areas and appears to be more pronounced in the region of the precuneus. This might have implications in clinical scanning. A specific pattern of regional cerebral glucose hypometabolism is seen in AD characteristically involving the precuneus (Minoshima et al. 1995). Reiman et al. (2004) demonstrated similar patterns of regional glucose hypometabolism in cognitively normal patients at risk of late-onset AD (i.e. carriers of the apolipoprotein E e4 allele) several decades before the onset of symptoms and structural changes on anatomic imaging. However, assessing the uptake in the precuneus by normalising the uptake in the patient’s brain and comparing it to a standard normal database has been complicated by studies that suggest that hyperglycaemia suppresses the uptake of ^18^F-FDG in the precuneus to a greater degree than in other regions of the brain (Ishibashi et al. 2017). This would mean that when normalisation of ^18^F-FDG uptake using whole brain or cerebellum is used in the context of hyperglycaemia during comparison of brain uptake with normal databases, the precuneus may demonstrate an artefactual reduction in uptake potentially leading to a false positive diagnosis of AD. Our study strongly supports these previous findings that the measured reduction in brain ^18^F-FDG uptake related to increasing BGL is more marked in the precuneus than it is in the whole brain or the cerebellum. In addition, we have found that elevated serum ketone levels induce a similar difference in regional effect on ^18^F-FDG uptake, with the degree of suppression related to ketones appearing more marked in the precuneus than in the whole brain or cerebellum. This should also be taken into consideration when undertaking whole brain normalisation and comparison with a normal database, particularly when assessing for early Alzheimer’s disease.

There are several limitations to our study. Patients were provided with detailed information on the prescribed preparatory diet and the importance of strict adherence; however, there was inevitable variability in the quantities of glucose/carbohydrates consumed and degrees of dietary adherence. Interestingly, the patients who reported failure to adhere strictly to the diet were all in ketosis at the time of their PETs, while all the patients who were not ketotic reported strict dietary adherence. Of the twelve (23%) patients not in ketosis, five were diabetic including the single patient with T1DM, with two patients on regular insulin. Nonetheless, this has meant that we have a relatively large range of BGL and serum ketone levels to examine and does not impact our findings of a negative association between ^18^F-FDG uptake, and serum glucose and ketone concentrations.

We included both non-diabetic (*n* = 39, 75%) and diabetic (*n* = 13, 25%) patients in our study, with five (10%) of the diabetic patients being on regular insulin. Insulin is known to impact the actions of certain subsets of GLUTs as well as the normal ketone response (Bickerton et al. 2008). Our patients were instructed to withhold insulin during their fasting period. Due to patient fasting and insulin being withheld prior to their PETs, we consider that all our patients would be in a low insulin state, but we did not measure insulin levels to confirm this. Furthermore, intracellular uptake of ^18^F-FDG in the brain is primarily driven by GLUT1 and, to a lesser extent, GLUT3, the actions of both being independent of insulin (Mochizuki et al. 2001; Avril 2004). Therefore, we believe that insulin levels should have a negligible impact on the cerebral ^18^F-FDG uptake seen in our study.

Twenty-seven patients (52%) were administered unfractionated heparin intravenously 15 min prior to ^18^F-FDG injection to increase serum free fatty acid (FFA) levels. Increased availability of FFA has been shown to increase rates of ketogenesis and serum ketones levels in a relatively short period of time after administration (i.e. < one hour) (Miles et al. 1983), more pronounced with unfractionated rather than low molecular weight heparin, although transient for doses in the range used in our cohort (Sasaki et al. 1994). While the administration of heparin is a potential confounder, we did not find a significant relationship between cerebral ^18^F-FDG uptake and heparin administration.

Patients may occasionally self-administer benzodiazepines when undergoing imaging studies to ameliorate symptoms of claustrophobia. Benzodiazepines can result in a global reduction in cerebral ^18^F-FDG uptake (Silva-Rodríguez et al. 2016). Given these studies were performed to assess for potential cardiac inflammation, we did not enquire about or record this information. It is possible that some of our cohorts may have self-administered benzodiazepines; however, there is no reason to believe that this will be more or less likely to be related to BGL or ketone levels and is likely to represent a random effect.

It is standard at our institution to perform cardiac inflammation PET/CTs with patients’ arms above their heads to reduce beam-hardening artefacts through the thorax and heart. A few patients in our cohort were scanned with their arms down due to physical limitations. While this has the potential to affect the accuracy of the measured cerebral SUVbw, the impact is felt likely minimal given that our analysis was performed on the attenuation-corrected PET images and with us evaluating whole brain SUVmean (corrected for bodyweight) and with the SUVmean extracted from relatively large brain regions, thereby reducing regional inconsistencies in measured uptake.

## Conclusion

Our study shows that increasing blood ketone levels may suppress ^18^F-FDG uptake in the entire brain, with this suppression being more marked in the region of the precuneus. To our knowledge, these effects of serum ketones have not previously been identified in the clinical scanning environment. Our study also provides further evidence to previous studies that show that possible suppression of ^18^F-FDG uptake in the brain due to increasing BGL is also more marked in the precuneus. Based on our findings, we suggest that when SUV cut-offs are used to determine whether patterns of ^18^F-FDG uptake fall within the physiologic or pathologic range the BGL and serum ketone levels should both be considered. In addition, given that the magnitude of this suppression of ^18^F-FDG uptake differs between brain regions (being more marked in the precuneus), this has implications for interpreting normalised brain SUVs when assessing for regional differences in brain metabolism using comparison with a normal database in the assessment of dementia.

## Data Availability

The data sets generated during and/or analysed during the current study are available from the corresponding author upon reasonable request.
